# Supplementary material for: Stochastic poromechanical analysis forecasts a notable exceedance probability for the 2017 Pohang, South Korea, Mw 5.5 earthquake
Source: Commun Earth Environ. 2026 Feb 7;7(1):236. doi: 10.1038/s43247-026-03268-7 (PMC12992117; doi:10.1038/s43247-026-03268-7)
Supplement: Supplementary file 2 — Supplementary Information [file 43247_2026_3268_MOESM2_ESM.pdf]

Supplementary Information for

Stochastic poromechanical analysis forecasts a notable exceedance probability for the 2017 Pohang, South Korea, M_w 5.5 earthquake

Haiqing Wu[1,2,3,*], Victor Vilarrasa[3], Francesco Parisio[3,4], Andrés Alcolea[5], Peter Meier[5], Jesus Carrera[2,4] and Maarten Saaltink[1,2]

[1] Department of Civil and Environmental Engineering (DECA), Universitat Politécnica de Catalunya (UPC), Barcelona, Spain
[2] Associated Unit: Hydrogeology Group (UPC-CSIC), Barcelona, Spain
[3] Global Change Research Group (GCRG), IMEDEA, CSIC-UIB, Esporles, Spain
[4] Institute of Environmental Assessment and Water Research (IDAEA), Spanish National Research Council (CSIC), Barcelona, Spain
[5] Geo-Energie Suisse AG, Zürich, Switzerland

[*]Corresponding author. Email: haiqing.wu@csic.es, now at the Institute of Marine Sciences (ICM), Spanish National Research Council (CSIC), Barcelona, Spain

This PDF file includes:

Supplementary Figs. S1 to S14
Supplementary Tables S1 to S3
Supplementary References: 1 to 5

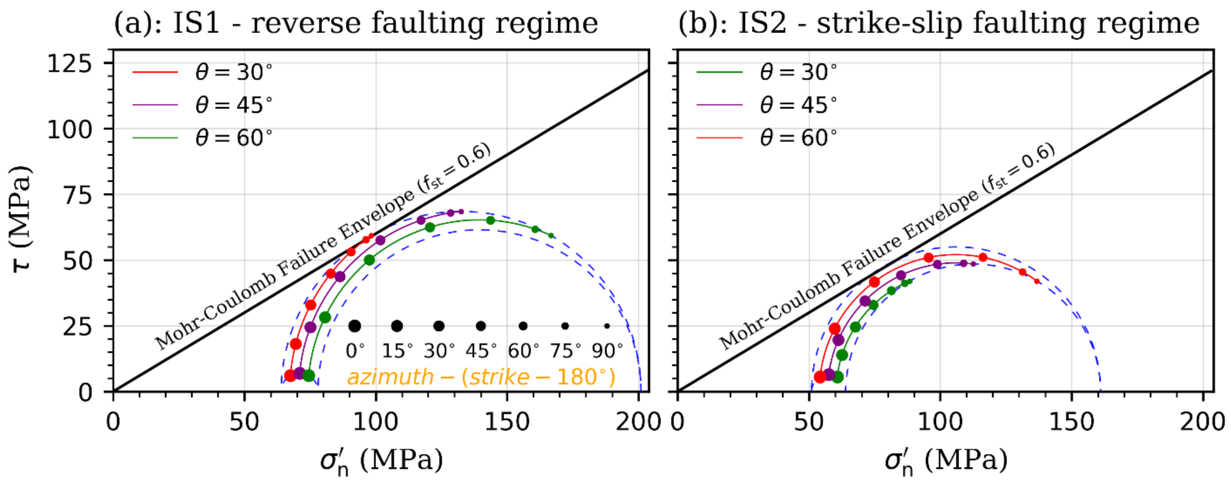

Supplementary Fig. S1. Mohr circles calculated with the in-situ stress states (a) IS1 (reverse faulting regime) and (b) IS2 (strike-slip faulting regime). The black solid lines are the Mohr-Coulomb failure envelopes assuming a static friction coefficient f_{st} of 0.6. The curves with seven size-scaled markers represent the trajectory of three planes with the dip angle of 30º, 45º and 60º and with varying the orientation from perpendicular to parallel to the maximum horizontal stress direction (azimuth). The size of markers is inversely proportional to the angle azimuth – (strike – 180°), as shown in (a). In the reverse faulting regime, an inclined fault with a low dip angle ($\leq(\pi/2\text{-atan} f_{st})/2 = 29.5°$ for $f_{st} = 0.6$) presents the most critical orientation when the fault strike is perpendicular to the azimuth of the maximum horizontal principal stress. Vertical faults represent the most critical dip angle in the strike-slip faulting regime, having the most critical orientation when the angle azimuth − (strike − 180°) equals $(\pi/2\text{-atan} f_{st})/2$, i.e., 29.5° for $f_{st} = 0.6$. Thus, the most critical fault dip and orientation in both regimes present the same correlation with the static friction coefficient, which is $(\pi/2\text{-atan} f_{st})/2$.

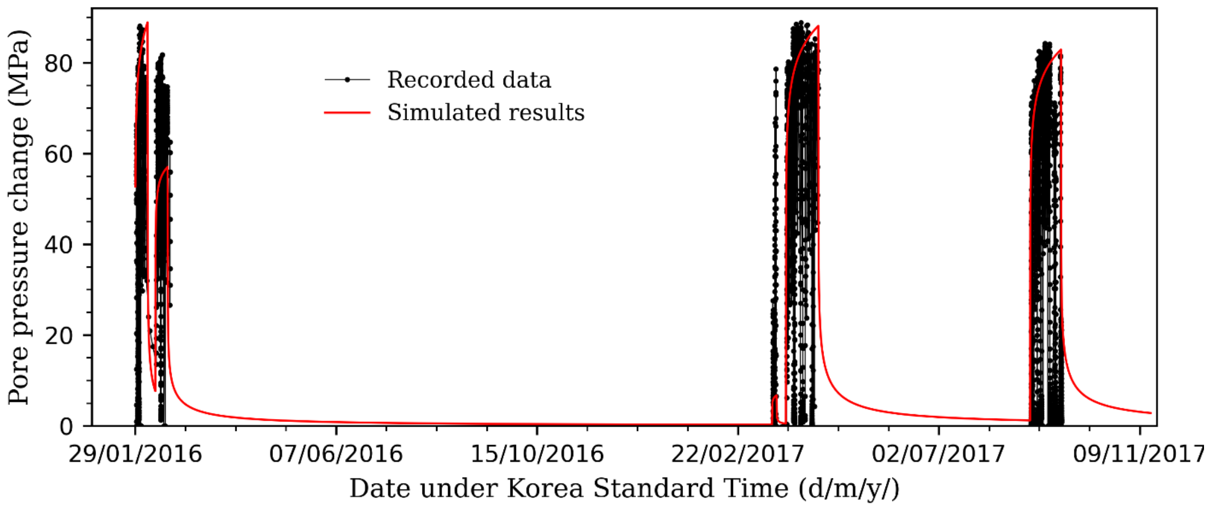

Supplementary Fig. S2. Comparison of temporal evolution of pore pressure buildup at the bottom hole of PX-2 between the simulated results and the recorded data from the beginning of the first hydraulic stimulation to the timestamp of the mainshock. The recorded data is taken from Yeo *et al.* [1]. The calibrated thickness of the pressurized region is 750 m.

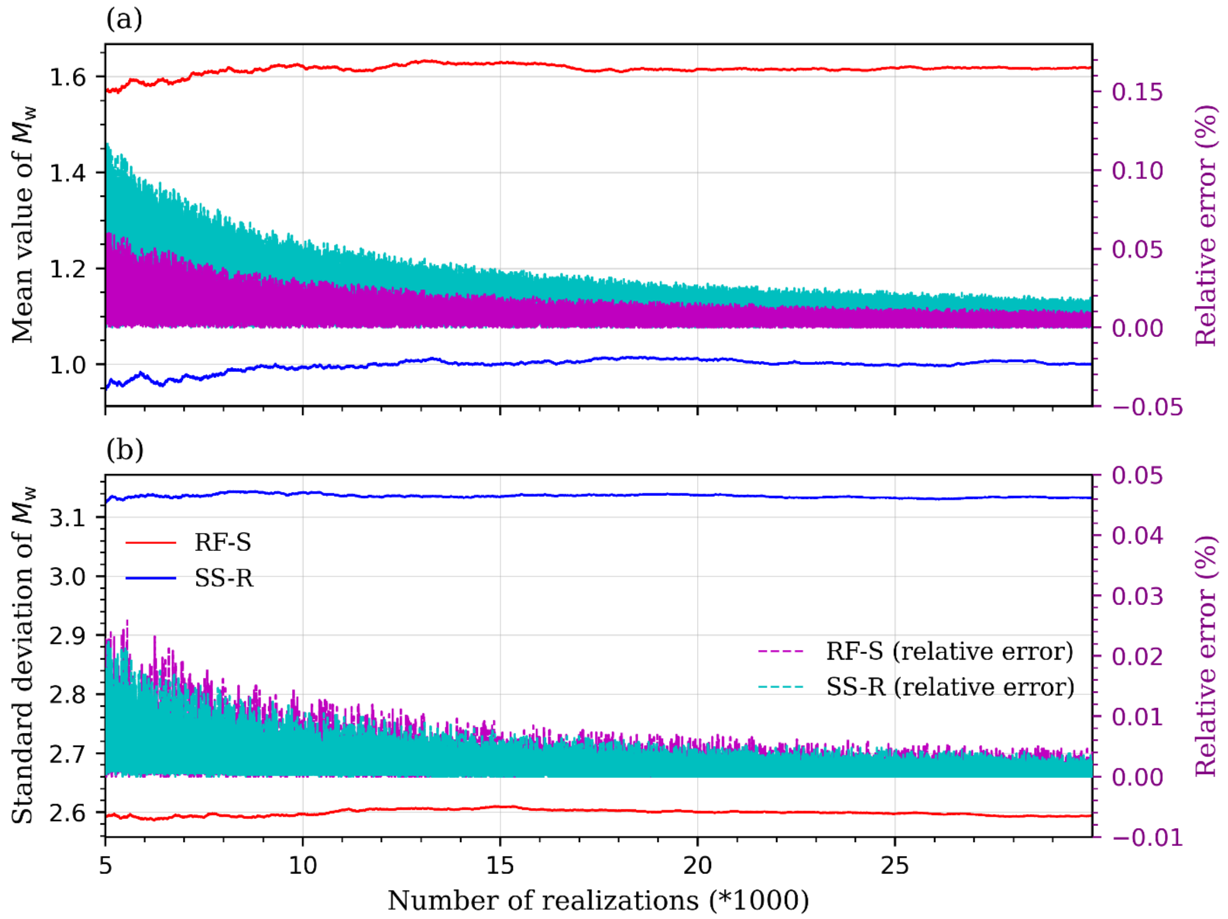

Supplementary Fig. S3. (a) Mean and (b) standard deviation of the earthquake magnitude M_w versus the number of realizations during Monte Carlo simulations for both RS-S and SS-R patterns. The relative error as a function of the number of realizations is also included, which refers to the right axis. It shows that both statistical metrics of M_w stabilize with increasing realizations for both slip patterns, reaching a stationary state after ~25,000 realizations, at which the relative errors are less than 0.03% and continue to asymptotically decay toward zero.

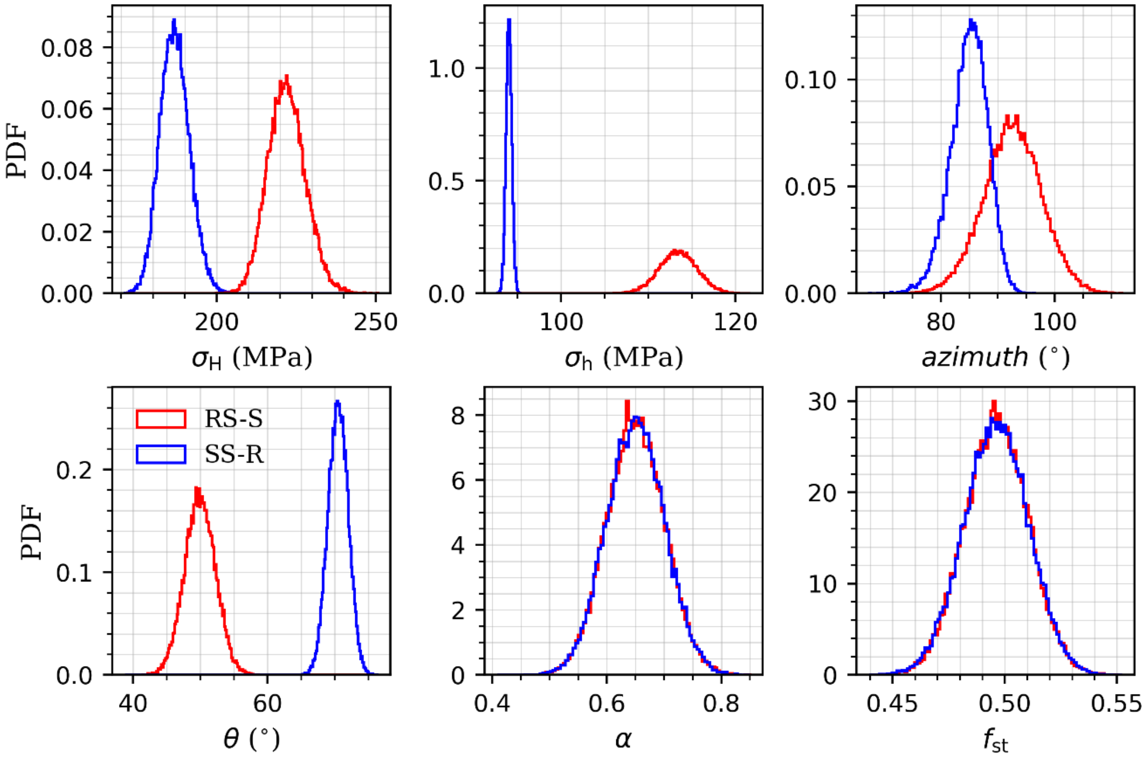

Supplementary Fig. S4. Probability density function (PDF) of random variables after the Monte Carlo simulations.

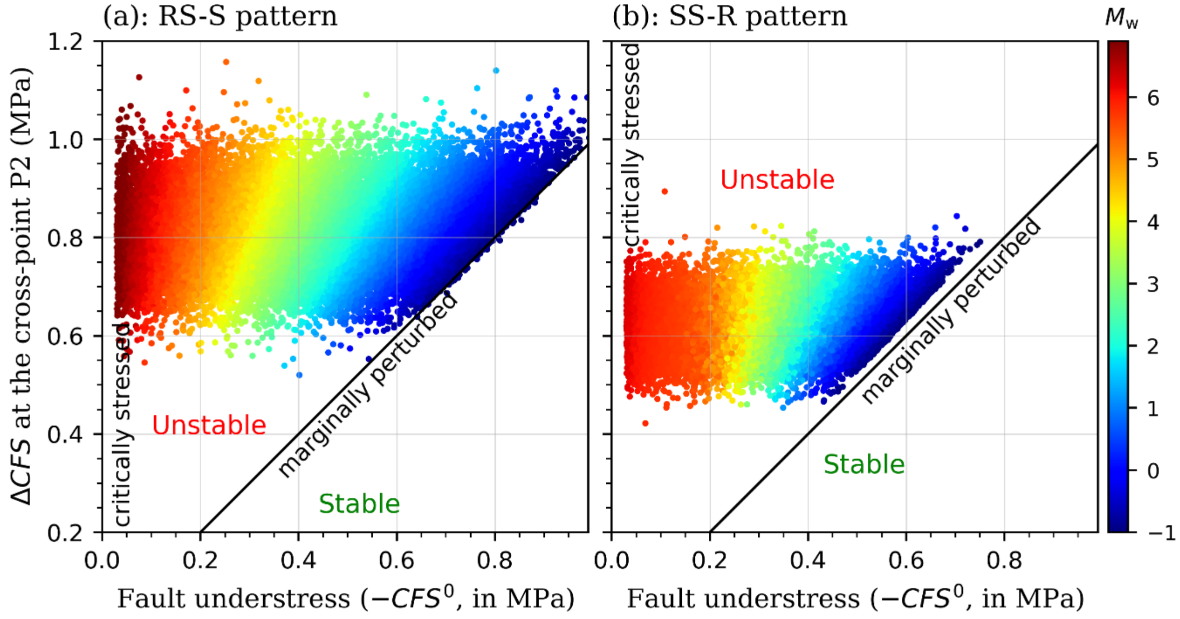

Supplementary Fig. S5. Fault stability and earthquake magnitude map conditioned to the *CFS*⁰ being within the interval of -1 to -0.03 MPa. Distribution of the Monte Carlo realizations in the parametric space with respect to the fault understress (-CFS^0) and the maximum change in fault stability (Δ*CFS* at the cross-point P2) for (a) the RS-S and (b) the SS-R patterns. Each dot depicts a single realization, defined by its CFS^0 and Δ*CFS* at P2, and colored by its moment magnitude (M_w).

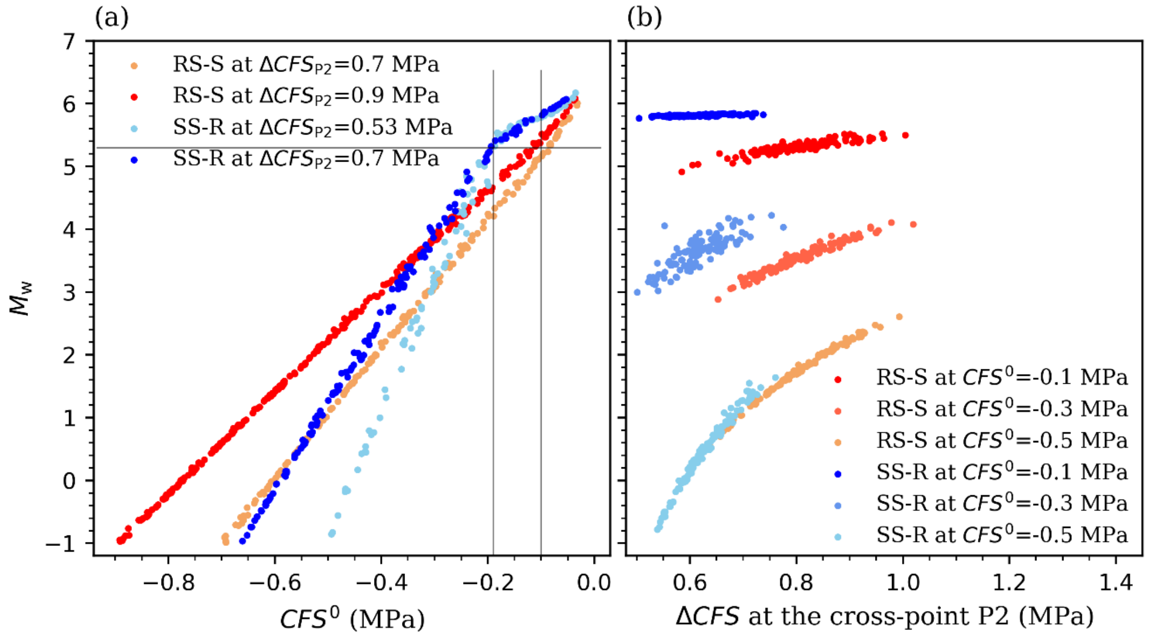

Supplementary Fig. S6. Quantitative scaling plot of the Monte Carlo simulations conditioned to the *CFS*⁰ being within the interval of -1 to -0.03 MPa. Earthquake magnitude M_w as a function of (a) the CFS^0 and (b) the Coulomb Failure Stress Change (Δ*CFS*) at the cross-point P2 for both RS-S and SS-R patterns. The dots imply the Monte Carlo realizations that are extracted from Fig. S5 at fixed values of Δ*CFS* at P2 in (a) and of CFS^0 in (b).

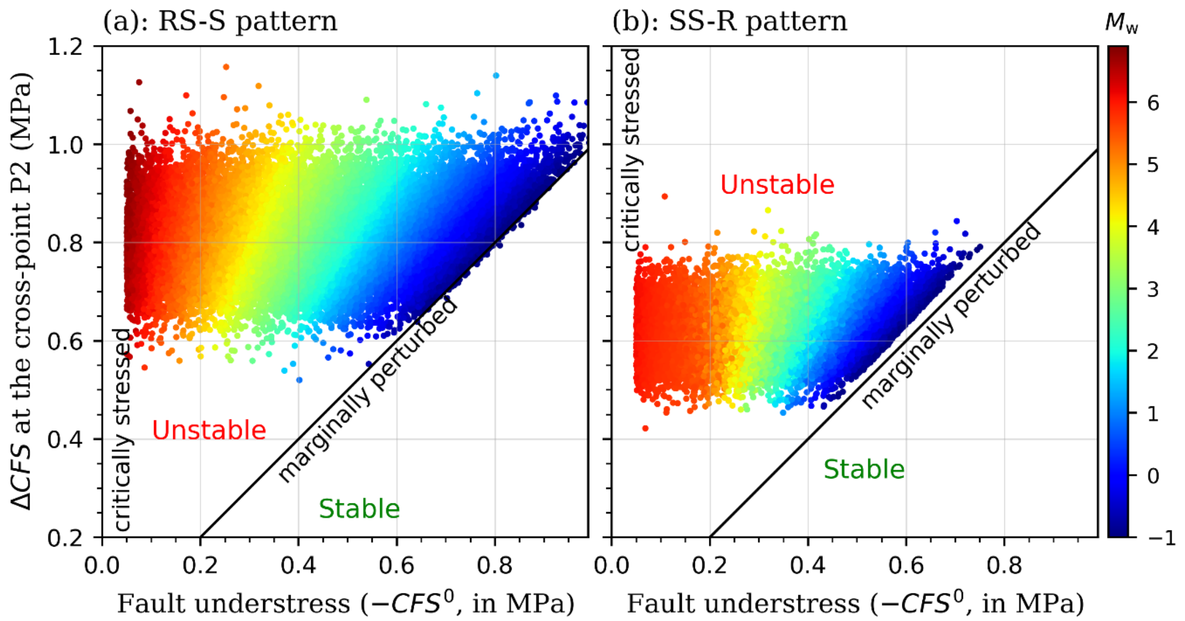

Supplementary Fig. S7. Fault stability and earthquake magnitude map conditioned to the CFS^0 being within the interval of -1 to -0.05 MPa. Distribution of the Monte Carlo realizations in the parametric space with respect to the fault understress (-CFS^0) and the maximum change in fault stability (ΔCFS at the cross-point P2) for (a) the RS-S and (b) the SS-R patterns. Each dot depicts a single realization, defined by its CFS^0 and ΔCFS at P2, and colored by its moment magnitude (M_w).

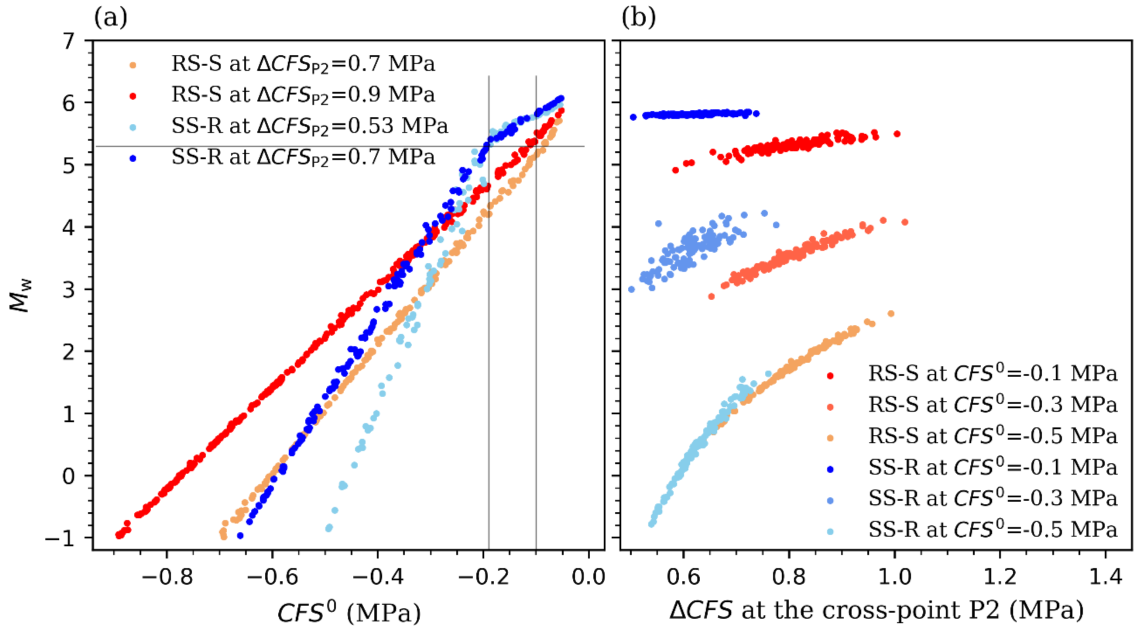

Supplementary Fig. S8. Quantitative scaling plot of the Monte Carlo simulations conditioned to the CFS^0 being within the interval of -1 to -0.05 MPa. Earthquake magnitude M_w as a function of (a) the CFS^0 and (b) the Coulomb Failure Stress Change (ΔCFS) at the cross-point P2 for both RS-S and SS-R patterns. The dots imply the Monte Carlo realizations that are extracted from Fig. S7 at fixed values of ΔCFS at P2 in (a) and of CFS^0 in (b).

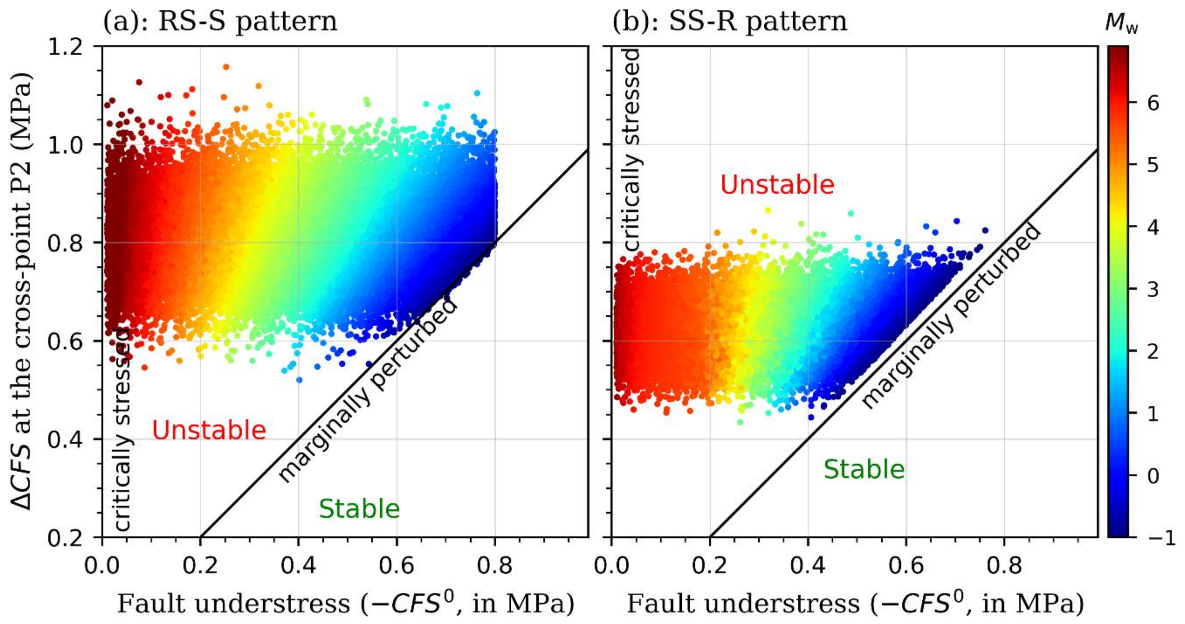

Supplementary Fig. S9. Fault stability and earthquake magnitude map conditioned to the CFS^0 being within the interval of -0.8 to -0.01 MPa. Distribution of the Monte Carlo realizations in the parametric space with respect to the fault understress ($-CFS^0$) and the maximum change in fault stability (ΔCFS at the cross-point P2) for (a) the RS-S and (b) the SS-R patterns. Each dot depicts a single realization, defined by its CFS^0 and ΔCFS at P2, and colored by its moment magnitude (M_w).

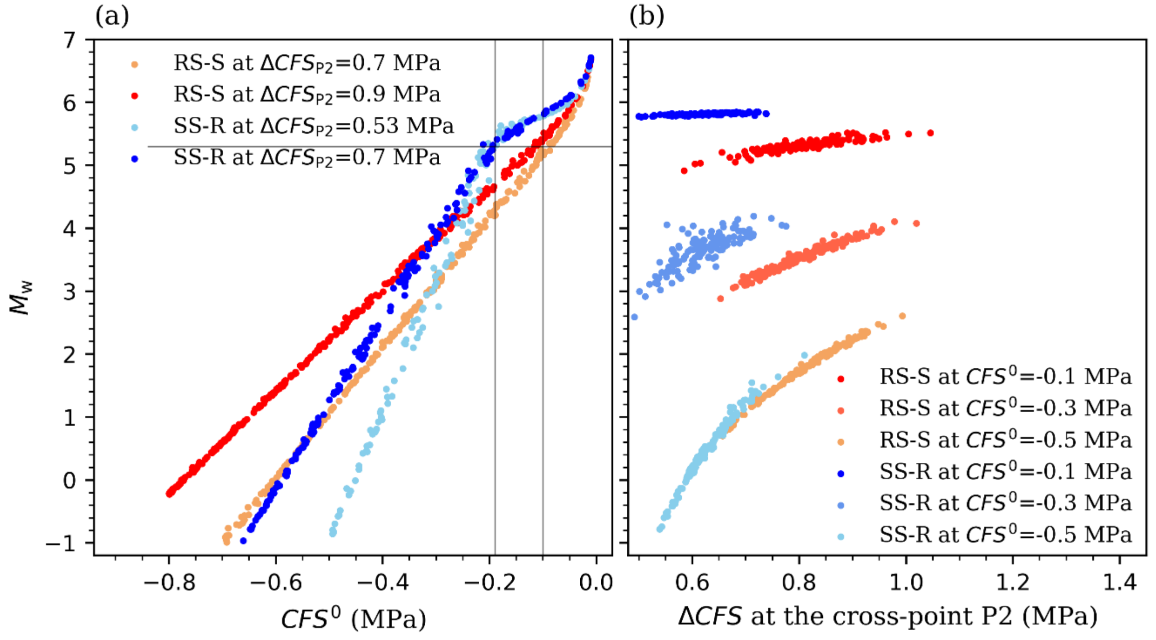

Supplementary Fig. S10. Quantitative scaling plot of the Monte Carlo simulations conditioned to the CFS^0 being within the interval of -0.8 to -0.01 MPa. Earthquake magnitude M_w as a function of (a) the CFS^0 and (b) the Coulomb Failure Stress Change (ΔCFS) at the cross-point P2 for both RS-S and SS-R patterns. The dots imply the Monte Carlo realizations that are extracted from Fig. S9 at fixed values of ΔCFS at P2 in (a) and of CFS^0 in (b).

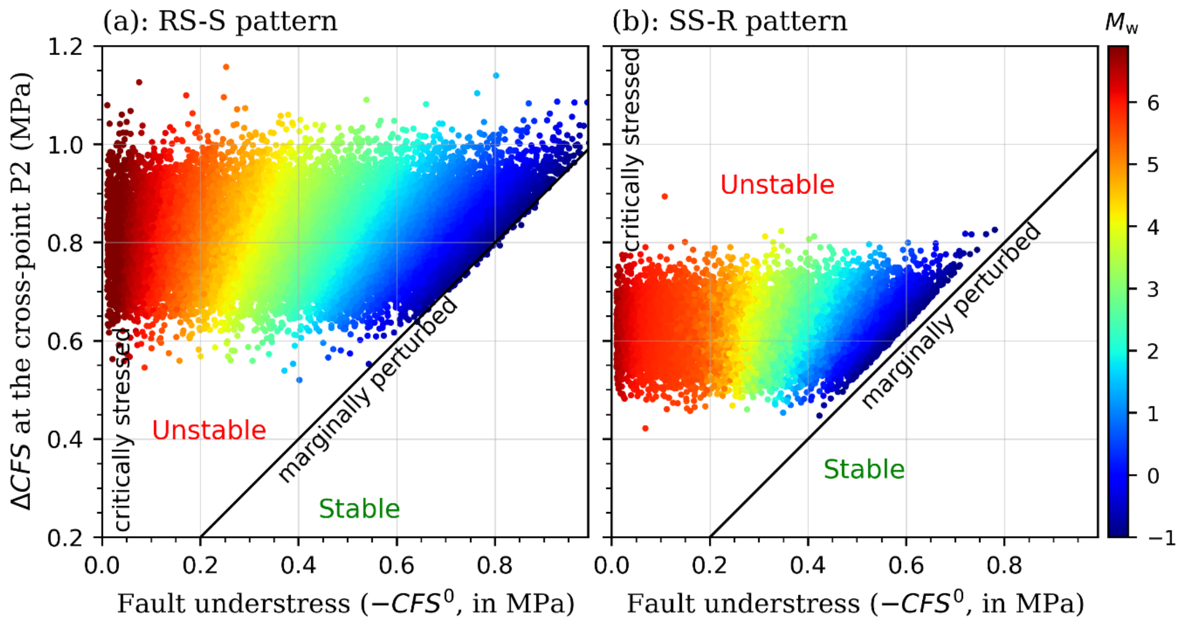

Supplementary Fig. S11. Fault stability and earthquake magnitude map conditioned to the CFS^0 being within the interval of -1.2 to -0.01 MPa. Distribution of the Monte Carlo realizations in the parametric space with respect to the fault understress (-CFS^0) and the maximum change in fault stability (ΔCFS at the cross-point P2) for (a) the RS-S and (b) the SS-R patterns. Each dot depicts a single realization, defined by its CFS^0 and ΔCFS at P2, and colored by its moment magnitude (M_w).

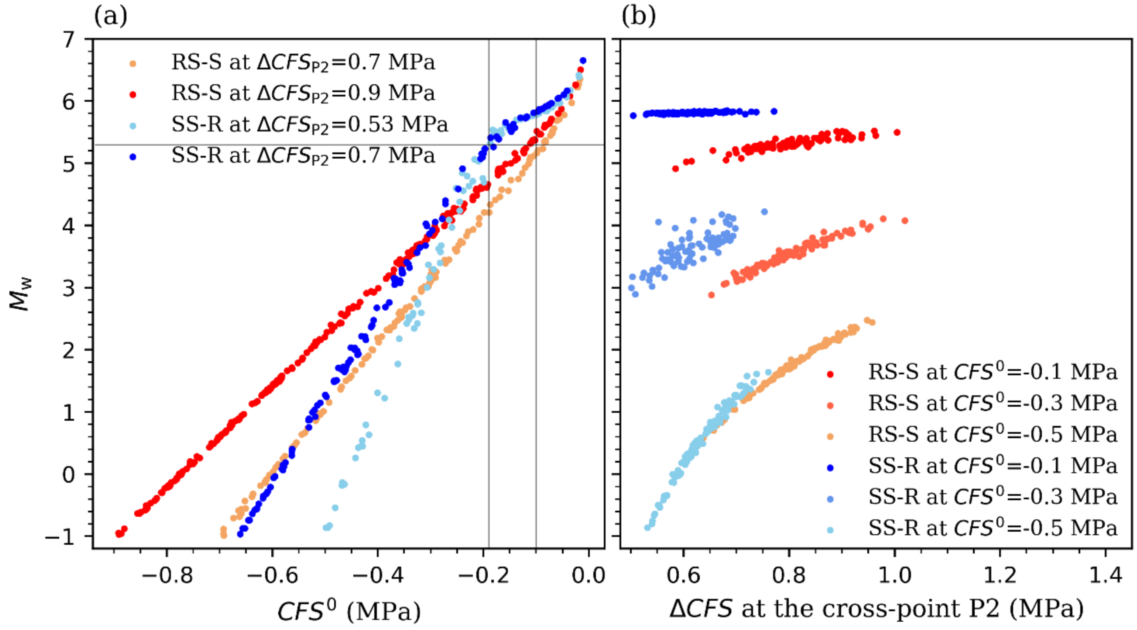

Supplementary Fig. S12. Quantitative scaling plot of the Monte Carlo simulations conditioned to the CFS^0 being within the interval of -1.2 to -0.01 MPa. Earthquake magnitude M_w as a function of (a) the CFS^0 and (b) the Coulomb Failure Stress Change (ΔCFS) at the cross-point P2 for both RS-S and SS-R patterns. The dots imply the Monte Carlo realizations that are extracted from Fig. S11 at fixed values of ΔCFS at P2 in (a) and of CFS^0 in (b).

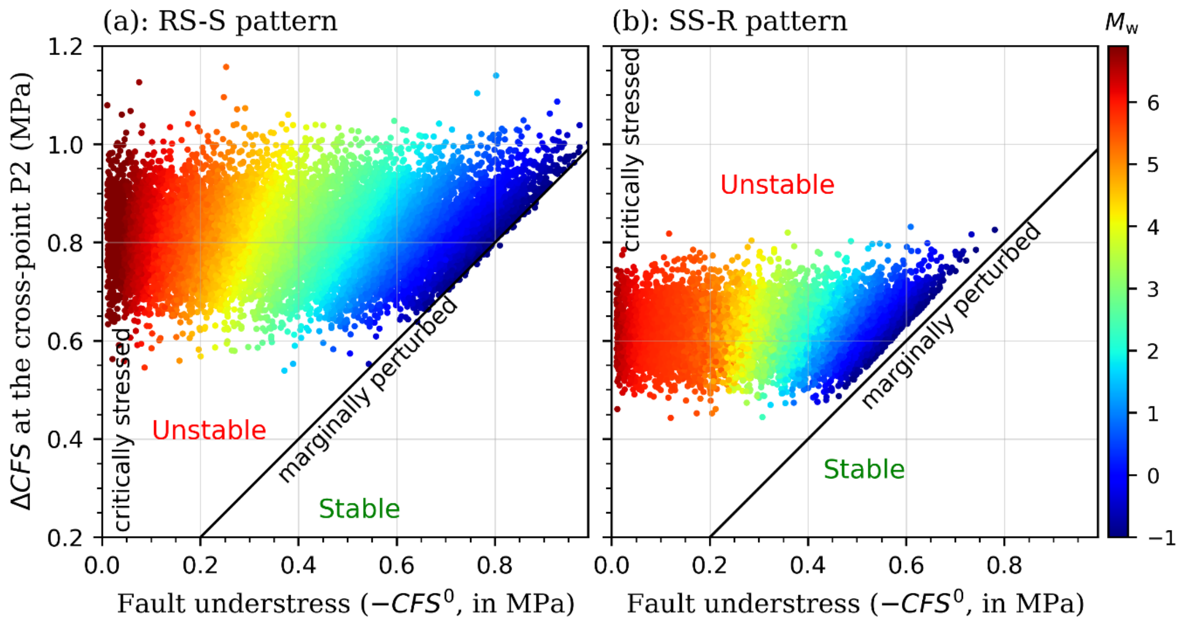

Supplementary Fig. S13. Fault stability and earthquake magnitude map conditioned to the *CFS^0* being within the interval of -2 to -0.01 MPa. Distribution of the Monte Carlo realizations in the parametric space with respect to the fault understress (-CFS^0) and the maximum change in fault stability (ΔCFS at the cross-point P2) for (a) the RS-S and (b) the SS-R patterns. Each dot depicts a single realization, defined by its CFS^0 and ΔCFS at P2, and colored by its moment magnitude (M_w).

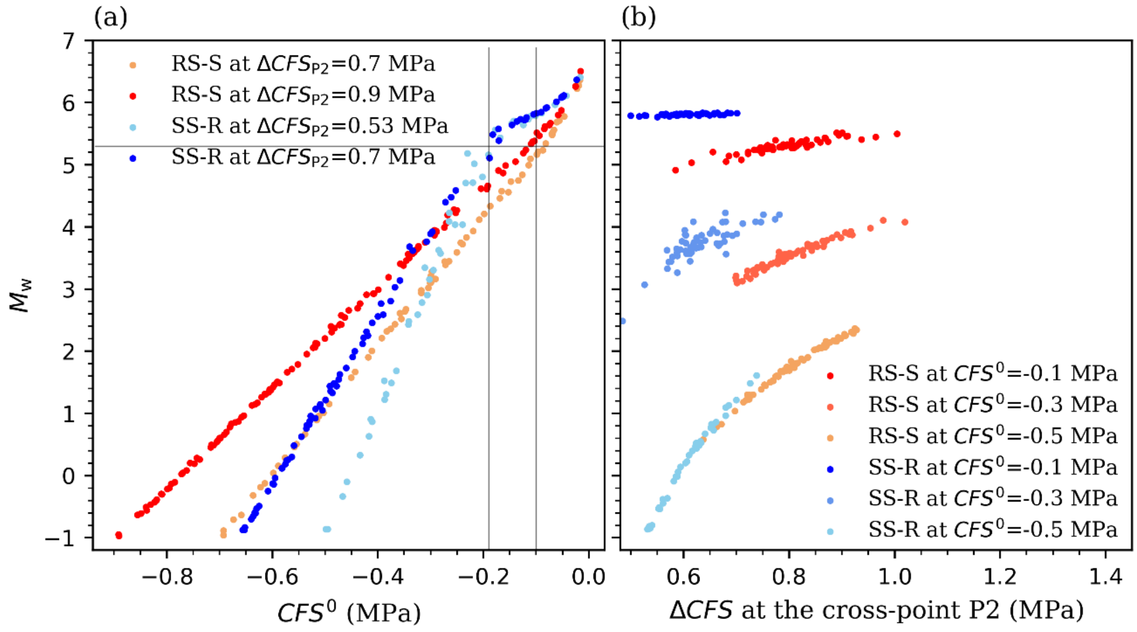

Supplementary Fig. S14. Quantitative scaling plot of the Monte Carlo simulations conditioned to the *CFS^0* being within the interval of -2 to -0.01 MPa. Earthquake magnitude M_w as a function of (a) the CFS^0 and (b) the Coulomb Failure Stress Change (ΔCFS) at the cross-point P2 for both RS-S and SS-R patterns. The dots imply the Monte Carlo realizations that are extracted from Fig. S13 at fixed values of ΔCFS at P2 in (a) and of CFS^0 in (b).

Supplementary Table S1. Plausible range of the in-situ stress and fault orientation constrained from plausibility analysis for both RS-S and SS-R patterns.

Parameter	Physical meaning	Minimum		Maximum		Mean Value		Unit
		RS-S	SS-R	RS-S	SS-R	RS-S	SS-R	
σ_H	The maximum horizontal principal stress	198	168	243	203	220.5	185.5	MPa
σ_h	The minimum horizontal principal stress	107	93	120	95	113.5	94	MPa
Azimuth	Azimuth of σ_H	77	74	111	100	N94	N87	°
θ	Fault dip	43	66	58	75	50.5	70.5	°
strike	Fault strike	214	221	215	225	214.5	223	°

Supplementary Table S2. Injection scheme used in the model, including five injection/shut-in periods in well PX-2 for analytical hydraulic modeling, which is simplified from the originally recorded data[1] of the three hydraulic stimulations in PX-2.

Number of cycles	Start date	End date	Duration (Day)	Mean injection rate (L/s)	Operation
1	29/01/2016	05/02/2016	8	1.87	Injection
	06/02/2016	10/02/2016	5	0	Shut-in
2	11/02/2016	18/02/2016	8	1.12	Injection
	19/02/2016	15/03/2017	391	0	Shut-in
3	16/03/2017	18/03/2017	3	0.15	Injection
	19/03/2017	24/03/2017	6	0	Shut-in
4	25/03/2017	14/04/2017	21	1.7	Injection
	15/04/2017	29/08/2017	137	0	Shut-in
5	30/08/2017	18/09/2017	20	1.59	Injection
	19/09/2017	15/11/2017	58	0	Shut-in

Supplementary Table S3. Other hydraulic and mechanical parameters of the reservoir in the Pohang EGS site.

Parameter	Physical meaning	Value	Unit	References
ϕ	Rock porosity	0.05	-	2, 3
k	Intrinsic permeability	5	μD	1, 4
φ	Fluid viscosity	0.2	mPa·s	1, 4
c_t	Total compressibility	9E-10	Pa^{-1}	1, 4
r_w	Radius of well PX-2	0.108	m	4
μ	Shear modulus	13.8	GPa	2
ν	Poisson's ratio	0.21	-	
p^0	Initial pore pressure	42	MPa	5

Supplementary References

1. Yeo, I. W., Brown, M. R. M., Ge, S. & Lee, K. K. Causal mechanism of injection-induced earthquakes through the Mw 5.5 Pohang earthquake case study. *Nat. Commun*. 11, 2614 (2020).
2. Kwon, S. et al. Characterization of 4.2-km-Deep Fractured Granodiorite Cores from Pohang Geothermal Reservoir, Korea. *Rock Mech. Rock Eng*. 52, 771-782 (2019).
3. Park, S. et al. First Hydraulic Stimulation in Fractured Geothermal Reservoir in Pohang PX-2 Well. *Procedia Eng*. 191, 829-837 (2017).
4. Hofmann, H. et al. First field application of cyclic soft stimulation at the Pohang Enhanced Geothermal 793 System site in Korea. *Geophys. J. Int*. **217**, 926-949 (2019).
5. Lee, K. K. Final Report of the Korean Government Commission on Relations between the 2017 Pohang Earthquake and EGS Project. https://doi.org/10.22719/KETEP- 2019043001 (2019).
